# Desuccinylation of pyruvate kinase M2 by SIRT5 contributes to antioxidant response and tumor growth

**DOI:** 10.18632/oncotarget.14346

**Published:** 2016-12-28

**Authors:** Ye Xiangyun, Niu Xiaomin, Gu linping, Xu Yunhua, Li Ziming, Yu Yongfeng, Chen Zhiwei, Lu Shun

**Affiliations:** ^1^ Department of Oncology, Shanghai Chest Hospital, Shanghai Jiaotong University, Shanghai 200030, China

**Keywords:** PKM2, succinylation, SIRT5, ROS, tumor growth

## Abstract

Tumor cells trends to express high level of pyruvate kinase M2 (PKM2). The inhibition of PKM2 activity is needed for antioxidant response by diverting glucose flux into the pentose phosphate pathway and thus generating sufficient reducing potential. Here we report that PKM2 is succinylated at lysine 498 (K498) and succinylation increases its activity. SIRT5 binds to, desuccinylates and inhibits PKM2 activity. Increased level of reactive oxygen species (ROS) decreases both the succinylation and activity of PKM2 by increasing its binding to SIRT5. Substitution of endogenous PKM2 with a succinylation mimetic mutant K498E decreases cellular NADPH production and inhibits cell proliferation and tumor growth. Moreover, inhibition of SIRT5 suppresses tumor cell proliferation through desuccinylation of PKM2 K498. These results reveal a new mechanism of PKM2 modification, a new function of SIRT5 in response to oxidative stress which stimulates cell proliferation and tumor growth, and also a potential target for clinical cancer research.

## INTRODUCTION

In contrast to normal proliferating cells, tumor cells have to survive in environments with varying oxygen and nutrient supplies [1, 2]. These circumstances make special demands upon the metabolism of tumor cells [3, 4]. An important molecular feature of tumor metabolism is the expression of glycolysis enzyme pyruvate kinase isoform M2 (PKM2) [5, 6], which catalyzes the transfer of phosphate from phosphoenolpyruvate (PEP) to ADP, resulting in the formation of pyruvate and ATP. In contrast to mitochondrial respiration, energy production by pyruvate kinase is independent of oxygen and allows survival of the cells under conditions of low oxygen supply. In addition, glycolytic intermediates are necessary as precursors for the synthesis of cell components. Inhibition of PKM2 accumulates glycolytic intermediates and promotes macromolecular biosynthesis and tumor growth [7–9]. The activity of PKM2 can be regulated by several post-translational modification: phosphorylation [10], acetylation [11] and oxidation [12], all of which are very important for tumor growth.

Sirtuins are a family of protein deacetylases which catalyze the nicotinamide NAD+ dependent removal of acetyl groups from modified lysine in substrates [13, 14]. SIRT5 is a member of Sirtuins family [15], but, interestingly, SIRT5 lacks Lys deacetylase activity and its physiological function has long been obscure. Recently, Park et al. identified SIRT5 as the enzyme catalyzing Lys desuccinylation. They also identified 779 putatively succinylated proteins, including PKM2.

Here, we report that PKM2 is succinylated at lysine 498 (K498) and succinylation increases its activity. SIRT5 binds to, desuccinylates and inhibits PKM2 activity. Increased levels of reactive oxygen species (ROS) decreases succinylation and activity of PKM2 by increasing its binding to SIRT5. Substitution of endogenous PKM2 with a succinylation mimetic mutant K498E decreases cellular NADPH production and inhibits cell proliferation and tumor growth. Moreover, inhibition of SIRT5 suppresses tumor cell proliferation through desuccinylation of PKM2 K498. These results reveal a new mechanism of PKM2 modification, a new function of SIRT5 in response to oxidative stress which stimulates cell proliferation and tumor growth, and also a potential target for clinical cancer research.

## RESULTS

### SIRT5 binds to and desuccinylates PKM2 at K498

**Figure 1:**
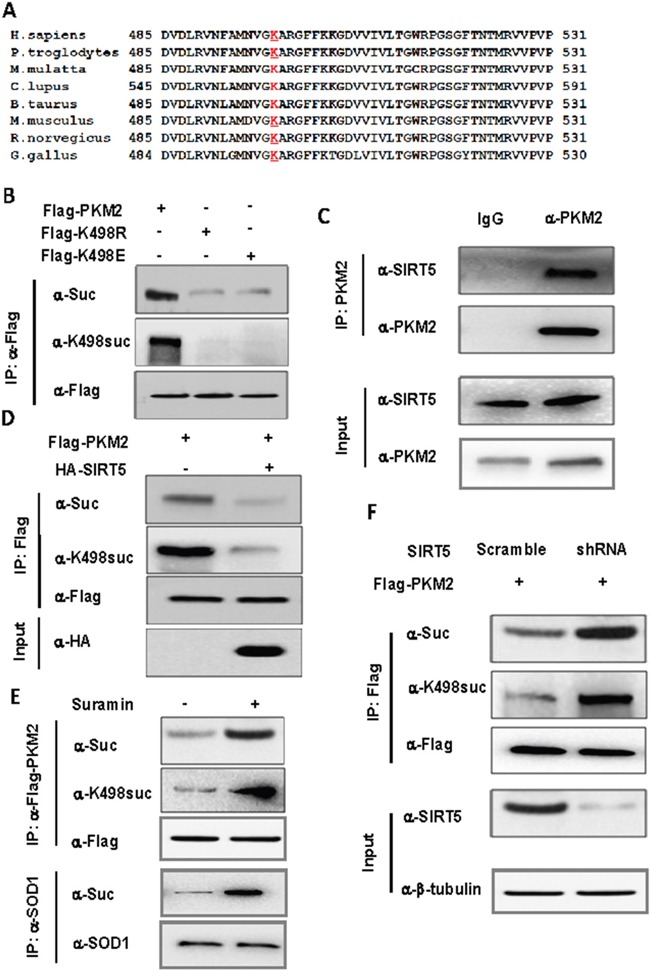
SIRT5 binds to and desuccinylates PKM2 at K498 **A**. Sequence alignment of PKM2 surrounding K498 from various species. K498 is underlined and marked red. **B**. K498 is the primary succinylation site of PKM2. The indicated plasmids were transfected into 293T cells and proteins were immunoprecipitated, followed by western blot for succinylation analysis. **C**. Endogenous SIRT5 binds to PKM2. A549 cells were lysed and endogenous PKM2 was immunoprecipitated. The binding of PKM2 to SIRT5 was examined by western blot. **D**. SIRT5 over expression decreases PKM2 succinylation. Succinylation levels of PKM2 in HEK293T cells expressing indicated plasmids were determined by western blot. **E**. Suramin, the SIRT5 inhibitor, increases the succinylation of PKM2. 293T cells were cultured in medium with or without Suramin (40 uM). The succinylation levels of PKM2 were determined by western blot. **F**. Knocking down SIRT5 increases succinylation level of PKM2. 293T cells were infected with retrovirus targeting SIRT5 and the levels of PKM2 protein and succinylation were determined by western blot. SIRT5 knockdown efficiency was determined by western blot.

Protein function is often regulated by diverse posttranslational modifications, such as phosphorylation, ubiquitination and acetylation. Recently, a systematic study of mammalian succinylome identified 2,565 succinylation sites on 779 proteins [16], including PKM2. 7 putative succinylation sites of PKM2 was identified by mass spectroscopy and among these sites, only K498 succinylation level of PKM2 increased for 2.6 fold in SIRT5 knockout mice by absolute stoichiometry [16], indicating K498 maybe the major succinylation site of PKM2. We analyzed the conservation of K498 and found that this site is highly conserved during evolution (Figure 1A). To confirm whether PKM2 is succinylated, we mutated K498 to R (K498E) and E (K498E) and found that the mutation decreased the overall succinylation level of PKM2 by 52.6% (Figure 1B). Given that the succinylation level of PKM2 increased for 2.6 fold in SIRT5 knockout mice, SIRT5 may desuccinylate PKM2 directly. We determined the binding of PKM2 to SIRT5, and found that both exogenous and endogenous PKM2 indeed binds to SIRT5 *in vitro* (Supplementary Figure 1A and 1C). Moreover, over expression of SIRT5 decreased the level of K498 succinylation of PKM2 (Figure 1D). To provide endogenous evidence for PKM2 succinylation at K498, we generated the K498 site-specific succinylation antibody by using a human PKM2 peptide (succinylated at K498) as an antigen. After purifying the antibody with excess unmodified peptides and enriched with succinylated peptides, we verified the specificity of the anti-K498-Suc antibody by western blot. The anti-K498-Suc antibody readily detected a band in wild-type PKM2, but no band in K498 mutants PKM2 (Figure 1B), indicating this antibody recognizes K498suc specifically. We then examined the effect of over expression or knocking down/inhibitor of SIRT5 on K498suc level by both the normal and site specific succinylation antibody. Over expression of SIRT5 significantly reduced PKM2 succinylation level at K498 (Figure 1D), while both SIRT5 inhibitor and knocking down SIRT5 increase K498suc level of PKM2 at K498 (Figure 1E and 1F), demonstrating that SIRT5 is the desuccinylase of PKM2. Taking together, these results suggest that PKM2 is indeed succinylated at K498 and SIRT5 is the desuccinylase of PKM2.

### Succinylation at K498 increases PKM2 activity

**Figure 2:**
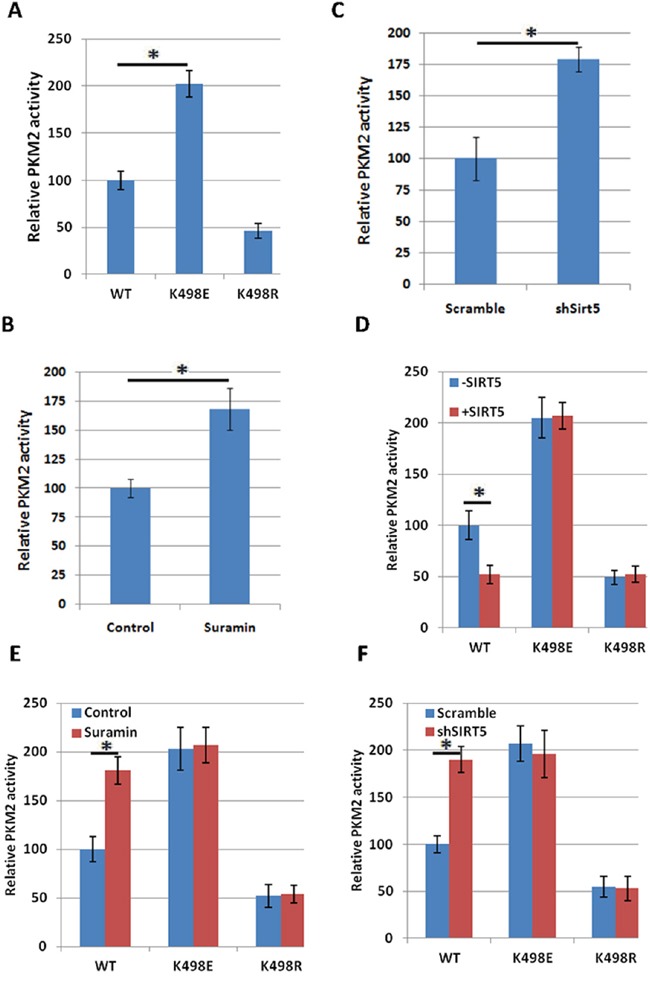
Succinylation at K498 increases PKM2 activity **A**. Succinylation mimetic mutation K498E increases PKM2 enzyme activity. Flag-tagged wild-type and mutant PKM2 proteins were expressed in 293T cells and purified by immunoprecipitation. PKM2 protein levels were analyzed by western blot in Supplementary Figure 2A. The relative enzyme activities of triplicate experiments ±SD are presented. Error bars represent ±SD for triplicate experiments. **B**. Inhibition of SIRT5 increases PKM2 activity. 293T cells were transfected with Flag-PKM2, followed by treatment with Suramin (40 uM). Flag-PKM2 was immunoprecipitated and activity was measured. PKM2 protein levels were analyzed by western blot in Supplementary Figure 2B. Relative enzyme activities of triplicate experiments ±SD are presented. **C**. Knocking down SIRT5 increases enzyme activity of PKM2. Flag-PKM2 was transfected into scramble and SIRT5 knocking down 293T cells. Flag-PKM2 was immunoprecipitated and activity was measured. SIRT5 knockdown efficiency in 293T cells was determined by q-PCR in Supplementary Figure 2C. Relative enzyme activities of triplicate experiments ±SD are presented. **D**. SIRT5 over expression decreases the activity of wild-type, but not K498E and K498R mutants of PKM2. PKM2 wild-type or mutants were co-expressed in 293T cells with or without SIRT5 respectively and purified with Flag beads, followed by enzyme assay. PKM2 and SIRT5 protein levels were analyzed by western blot in Supplementary Figure 2D. The mean value of triplicates and standard deviation (±SD) are presented. **E**. Suramin increases the activity of wild-type, but not K498E and K498R mutants of PKM2. PKM2 wild-type or mutants were transfected into 293T cells with or without the treatment of Suramin (40 uM). Proteins were purified with Flag beads, followed by enzyme assay. PKM2 protein levels were analyzed by western blot in Supplementary Figure 2E. The mean value of triplicates and standard deviation (±SD) are presented. **F**. SIRT5 knocking down increases the activity of wild-type, but not K498E and K498R mutants of PKM2. PKM2 wild-type or mutants were transfected into scramble and SIRT5 knocking down 293T cells respectively and proteins were purified with Flag beads, followed by enzyme assay. PKM2 protein levels were analyzed by western blot in Supplementary Figure 2F. The mean value of triplicates and standard deviation (±SD) are presented.

To test the effect of succinylation on PKM2 activity, we transfected 293T cells with PKM2 wild-type or K498E succinylation mimetic mutant or K498R succinylation resistant mutant, immunopurified the proteins and measured their activity. We found that the mutation of K to succinylation mimetic E increases PKM2 activity about 2.2 fold (Figure 2A and Supplementary Figure 2A), indicating that succinylation at K498 may increase PKM2 activity. Therefore, SIRT5 may act as a negative regulator of PKM2 activity. To test this hypothesis, we examined the function of SIRT5 in regulation of PKM2 enzyme activity. First, we immunopurified ectopically expressed Flag-PKM2 from 293T cells treated with SIRT5 inhibitors Suramin and measured the activity. Consistent with the hypothesis, treatment of cells with Suramin increased the activity of PKM2 (Figure 2B and Supplementary Figure 2B). Next, we measured the activity of PKM2 from control or SIRT5 knock down 293T cells, and found that knocking down of SIRT5 increased the activity of PKM2 approximately by 79% (Figure 2C and Supplementary Figure 2C). Conversely, over expression of SIRT5 reduced PKM2 activity, however, it had little effect on the activity of PKM2 K498E and K498R mutants (Figure 2D and Supplementary Figure 2D), suggesting that SIRT5 inhibits PKM2 mostly via desuccinylating K498. Furthermore, either inhibiting SIRT5 with Suramin or knocking down SIRT5 increased PKM2 activity (Figure 2E and Supplementary Figure 2E, 2F and Supplementary Figure 2F), but had no effect on the activity of PKM2 K498E and K498R mutants, providing further evidence supporting that SIRT5 inhibits PKM2 activity via desuccinylation K498.

### Succinylation at K498 of PKM2 sensitizes cells to oxidative damage

**Figure 3:**
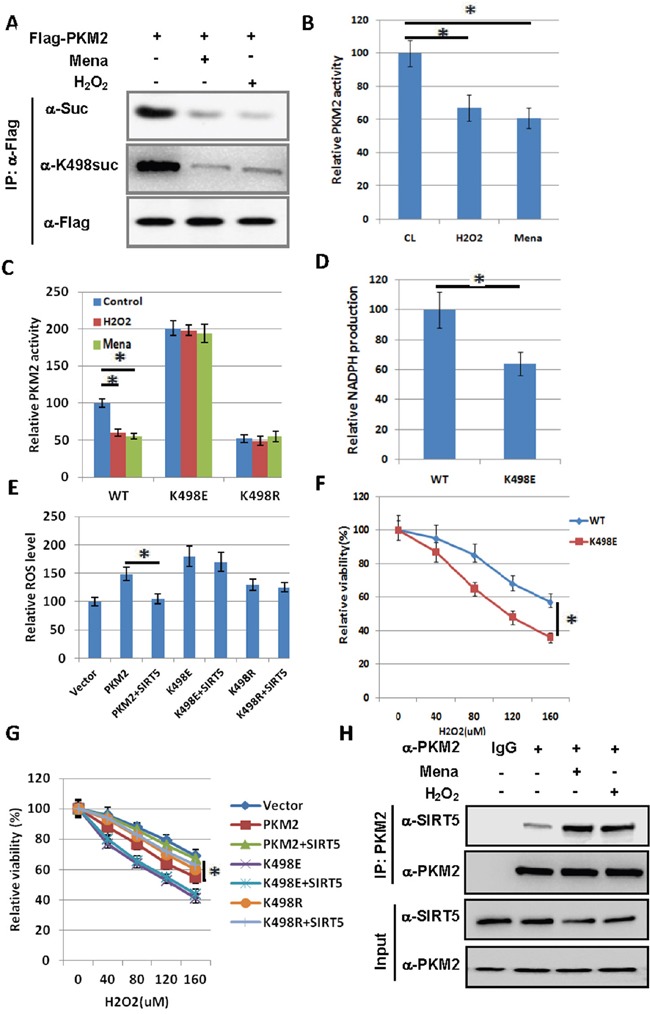
Succinylation at K498 of PKM2 sensitizes cells to oxidative damage **A**. H_2_O_2_ and Mena treatment decrease succinylation level of PKM2. 293T cells were transfected with Flag-PKM2 and treated with 500 uM H_2_O_2_ for 1h or 50uM Mena for 30min as indicated. The succinylation levels of PKM2 were determined by western blot. **B**. H_2_O_2_ and Mena induced oxidative stress decreases PKM2 activity. 293T cells were transfected with Flag-PKM2 and treated with 500 uM H_2_O_2_ for 1h or 50uM Mena for 30min, followed by immunoprecipitation and enzyme activity was measured and mean values of relative enzyme activity of triplicate experiments with standard deviation (±SD) are presented. **C**. H_2_O_2_ and Mena induced oxidative stress decreases wild-type PKM2 activity, but not K498E and K498R mutants. 293T cells were transfected with indicated plasmids, followed by treatment of 500 uM H_2_O_2_ for 1h or 50uM Mena for 30min, proteins were immunoprecipitated, and PKM2 activity was assayed. Relative enzyme activities of triplicate experiments ± SD are presented. **D**. A549 cells stably expressing succinylation mimetic K498E mutant reduces NADPH production. Stable cells verified in Figure 4A were prepared, and NADPH was measured using a NADPH kit. Error bars represent ±SD for triplicate experiments. **E**. The effect of over expression of PKM2 on ROS levels can be reversed by co-expression of SIRT5, but not on K498E and K498R mutants. 293T cells were transfected with indicated plasmids and DCF staining was performed to measure ROS levels. Error bars represent ±SD for triplicate experiments. PKM2 and SIRT5 protein levels were analyzed by western blot in Supplementary Figure 3A. **F**. Succinylation mimetic K498E mutant sensitizes cells to oxidative damage. A549 cells expressing PKM2 wild type or K498E mutant were exposed to different concentrations of H_2_O_2_ as indicated in the figure for 24h, and the viability of cells was measured by trypan blue exclusion. Error bars represent ±SD for triplicate experiments. **G**. SIRT5 can reverse the sensitization of cells to ROS caused by over expression of PKM2, but not K498E and K498R mutants. 293T cells were transfected with indicated plasmids and exposed to different concentrations of H_2_O_2_ as indicated in the figure for 24h. The viability of cells was measured by trypan blue exclusion. Error bars represent ±SD for triplicate experiments. **H**. H2O2 and Mena treatment increase the binding of PKM2 to SIRT5. 293T cells were treated with 500 uM H_2_O_2_ for 1h or 50uM Mena for 30min as indicated. The interaction between PKM2 and SIRT5 was determined by immunoprecipitation followed by western blot.

As Anastasiou et al. found that inhibition of PKM2 can divert glucose flux into the pentose phosphate pathway and thereby generate sufficient reducing potential to remove ROS [12], we treated cells with hydrogen peroxide (H_2_O_2_) or Mena to see whether oxidative stress can reduce PKM2 succinylation level and enzymatic activity. We observed that K498 succinylation level of PKM2 was decreased by H_2_O_2_ and Mena treatment in 239T cells (Figure 3A). We immunopurified ectopically expressed Flag-PKM2 from H_2_O_2_ or Mena treated cells and found that along with a reduction of succinylation, PKM2 activity was decreased about 40% upon H_2_O_2_ or Mena treatment (Figure 3B). Moreover, H_2_O_2_ or Mena treatment had no significant effect on the activity of PKM2 K498E and K498R mutants (Figure 3C), indicating that oxidative stress inhibited PKM2 activity via K498 desuccinylation.

**Figure 4:**
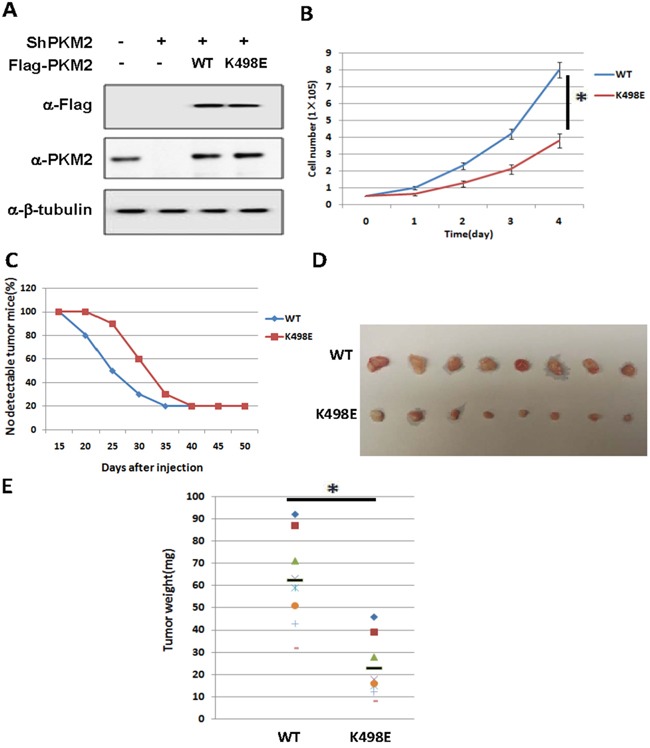
Succinylation at K498 of PKM2 suppresses cell proliferation and tumor growth **A**. The expression of putting-back PKM2 wild type and K498E in endogenous PKM2 knocking-down A549 stable cell lines. Whole cell lysates were prepared from A549 stable cell lines as indicated, followed by western analysis. **B**. Succinylation mimetic mutant K498E suppresses cell proliferation. 5×10^4^ indicated stable cells were seeded in each well. Cell numbers were counted every 24 h. Error bars represent cell numbers ±SD for triplicate experiments. **C, D**. Succinylation mimetic mutant K498E inhibits xenograft tumor growth *in vivo*. Nude mice were injected with A549 cells expressing PKM2 wild-type or K498E mutant. The time of tumorigenesis was presented in (C). The xenograft tumors were dissected and measured after 7 weeks and shown in (D). **E**. Quantification of average weight of xenograft tumors is shown. Error bars represent ±SD for eight tumors.

To explore the physiological significance of PKM2 succinylation at K498, we established stable cell lines in A549 cells with knocking down of endogenous PKM2 and putting back of Flag-tagged WT or K498E mutant of PKM2 (Figure 4A), respectively, followed by the measurement of NADPH. We found that the NADPH level decreased 36% in A549 cells expressing PKM2 K498E mutant compared with cells expressing wild-type PKM2 (Figure 3D), suggesting SIRT5 mediated desuccinylation of PKM2 may play a role in antioxidation. To further corroborate the finding of the role of suscinylation in suppressing the function of PKM2 in ROS clearance, we checked the effect of ectopically expressed PKM2 and SIRT5 on endogenous ROS level. Over expression of PKM2 increased the ROS level by 50%, and co-expression of SIRT5 can reverse the effect of PKM2 over expression on ROS level, while co-expression of SIRT5 cannot reduce the ROS level increased by over expression of PKM2 K498E and K498R mutants (Figure 3E). Supporting the functional importance of K498 succinylation in suppressing cellular antioxidation response, A549 cells expressing PKM2 K498E mutant were much more sensitive to hydrogen peroxide than the cell expressing wild-type PKM2 (Figure 3F). As SIRT5 catalyzes K498 desuccinylation of PKM2, next we examined the oxidative stress response of PKM2 and SIRT5 over expressed 293T cells. We found that PKM2 over expressing cells are more sensitive to oxidative stress, co-expression of SIRT5 can desensitize these cells to ROS, while co-expression of SIRT5 cannot desensitize cells expressing PKM2 K498E and K498R mutants (Figure 3G). As K498 succinylation level of PKM2 was decreased by H_2_O_2_ and Mena treatment (Figure 3A), we also determined the effect of oxidative stress on the interaction between PKM2 and SIRT5 and found that H_2_O_2_ and Mena treatment increase the binding of PKM2 to SIRT5 (Figure 3H). Collectively, succinylation at K498 of PKM2 sensitizes cells to oxidative damage.

### Succinylation at K498 of PKM2 suppressed cell proliferation and tumor growth

Given the high expression of PKM2 in cancer cells and K498 succinylation increases PKM2 activity, we examined the effect of K498 succinylation of PKM2 on cell proliferation and tumor growth by PKM2 knocking-down and putting-back stable cell lines. Endogenous PKM2 knockdown efficiency and the exogenous PKM2 protein level compared to endogenous level were shown in Figure 4A. From the result, we can see the endogenous PKM2 knockdown efficiency is high and the PKM2 putting back level is similar to endogenous PKM2. We found that A549 cells ectopically expressing succinylation-mimetic PKM2 K498E proliferated slower than cells expressing wild-type PKM2, indicating that succinylation of K498 inhibits cell proliferation (Figure 4B). To further determine whether K498 succinylation of PKM2 inhibits tumor growth, we performed xenograft experiment in immunodeficient nude mice using the A549 stable cell lines described above. Five million cells expressing either PKM2 wild-type or K498E mutant were injected into nude mice subcutaneously, and tumors were dissected after around 7 weeks. We found that cells expressing PKM2 K498E developed tumors much slower than cells expressing wild-type PKM2 (Figure 4C), as determined by both tumor volume (Figure 4D) and tumor weight (Figure 4E). Thus, Succinylation at K498 of PKM2 suppressed cell proliferation and tumor growth.

### Inhibition of Sirt5 suppresses tumor cell proliferation through desuccinylation of PKM2 K498

**Figure 5:**
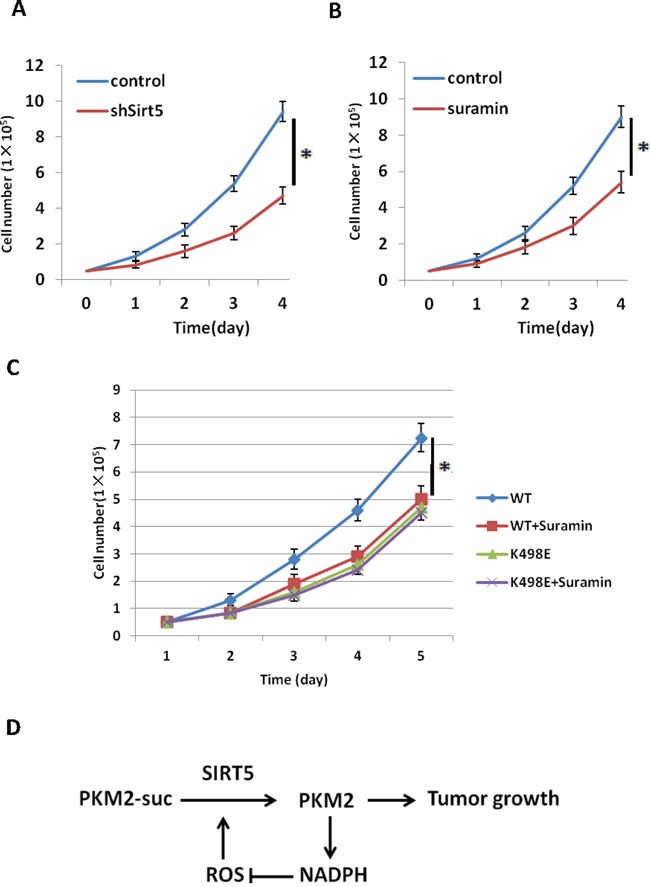
Inhibition of Sirt5 suppresses tumor cell proliferation through desuccinylation of PKM2 K498 **A**. Sirt5 knockdown suppresses cell proliferation. 5×10^4^ indicated A549 stable cells were seeded in each well. Cell numbers were counted every 24 h. Error bars represent cell numbers ±SD for triplicate experiments. SIRT5 knockdown efficiency in A549 cells was determined by q-PCR in Supplementary Figure 4A. **B**. Inhibition of SIRT5 suppresses cell proliferation. 5×10^4^ indicated A549 stable cells were seeded in each well, followed by treatment with Suramin (40 uM). Cell numbers were counted every 24 h. Error bars represent cell numbers ±SD for triplicate experiments. **C**. Inhibition of Sirt5 suppresses tumor cell proliferation through desuccinylation of PKM2 K498. A549 stable cell lines expressing PKM2 wild type and K498E were verified in Figure 4A and treated with Suramin (40 uM). Cell numbers were counted every 24 h. Error bars represent cell numbers ±SD for triplicate experiments. **D**. Working model for Sirt5 mediated PKM2 desuccinylation in response to oxidative stress to promote tumor growth and eliminate ROS.

To further prove SIRT5 mediated desuccinylation of PKM2 is important for tumor cell proliferation, we knocked down Sirt5 in A549 cells (Supplementary Figure 4A) and compared the cell proliferation rate with control cells. Result showed that knockdown Sirt5 significantly inhibits lung tumor cells proliferation (Figure 5A). To confirm this finding, we treated a549 cells with SIRT5 inhibitors Suramin and checked its effect on cell proliferation and found that Sirt5 inhibitor also suppressed tumor cell proliferation (Figure 5B), indicating Sirt5 is a potential target for lung tumor treatment. To demonstrate whether the effect of sirt5 inhibition on tumor growth is through PKM2 K498 succinylation, we carried out rescue experiments by treating previously described A549 stable cell lines expressing PKM2 wild type or K498E mutant (Figure 4A) with SIRT5 inhibitor Suramin. Result showed that Suramin can only inhibit tumor cells expressing PKM2 wild type, but had no effect on K498E expressing cells (Figure 5C), demonstrating inhibition of Sirt5 suppresses tumor cell proliferation through desuccinylation of PKM2 K498.

## DISCUSSION

Metabolic alteration has emerged as a growth advantage for cancer cells. In this study, we demonstrated that desuccinylation of PKM2 by SIRT5 inhibits its activity in response to oxidative stress. This inhibition of PKM2 may facilitate the redirection of glucose metabolites into the pentose phosphate shunt, producing sufficient NADPH to eliminate ROS (Figure 5D). This may be an adaptive response which protects the cells from oxidative stress, as NADPH is required to generate the reduced form of glutathione, which is a major intracellular defense against damage mediated by ROS. Our data indicate that PKM2 desuccinylation by SIRT5 would provide an advantage for tumor cell growth by allowing them to sustain antioxidant responses and thereby support cell survival and proliferation under acute oxidative stress. Another finding of this study is that inhibition of Sirt5 can suppress tumor cell proliferation through desuccinylation of PKM2 K498, which provides a potential target for clinical cancer research and treatment.

## MATERIALS AND METHODS

### Plasmid construction

Full-length cDNA of PKM2 was amplified by PCR and cloned into indicated vectors including pcDNA3-FLAG and pQCXIH, SIRT5 was cloned to pCDNA3-HA vector. Point mutations for PKM2 were generated by site-directed mutagenesis. The SIRT5 shRNA and scramble plasmids (Sigma) were commercially purchased.

### Cell culture and transfection

HEK293T were cultured in Dulbecco's modified Eagle's medium (Invitrogen) supplemented with 10% fetal bovine serum (HyClone), 100 units/ml penicillin and 100 μg/ml streptomycin (Gibco). Human lung carcinoma A549 cells were cultured in Nutrient Mixture F-12 Ham Kaighn's Modification (F12K) medium (Sigma) with 10% fetal bovine serum, 100 units/ml penicillin and 100 μg/ml streptomycin. Cell transfection was carried out by Lipofectamine 2000 according to the manufacturer's protocol (Invitrogen).

### Cell lysis, immunoprecipitation, immunoblotting and antibody

Cells were lysed in NP40 buffer containing 50mM Tris pH 7.5, 150mM NaCl, 0.3% Nonidet P-40, 1 μg/ml aprotinin, 1 μg/ml leupeptin, 1 μg/ml pepstatin, 1mM Na_3_VO_4_ and 1mM PMSF. Cell lysates were incubated with anti-Flag beads (Sigma) for 3 hr at 4°C, the beads were washed with NP-40 buffer three times, and then subjected to SDS-PAGE or eluted by Flag peptides for enzyme activity assay. Western blotting was performed according to standard protocol. Antibodies specific to Flag (Sigma), HA (Santa Cruz), PKM2 (Cell Signaling) and β-actin (Sigma) were commercially purchased.

### PKM2 enzyme activity assay

Pyruvate kinase activity was measured by a continuous assay coupled to lactate dehydrogenase (LDH). The change in absorbance resulting from NADH oxidation was measured using a F-4600 Fluorescence Spectrophotometer (HITACHI). Assays for PK activity were carried out as previously described [7].

### Cell treatment

Cultured cells were treated with Suramin, H_2_O_2_ or Mena at the concentration of 40 uM, 500 uM and 50 uM respectively for indicated time in figure legend.

### Measurement of intracellular ROS level

ROS production was determined by incubating the A549 stable cells in serum-free medium containing 10 μM fluorescent dye 2’,7’-dichlorofluorescein diacetate (DCF, Sigma) at 37°C for 30 min, washing by serum-free medium for three times, followed by fluorescence analysis.

### Establishment of knocking-down and putting-back stable cell lines

All retroviruses were produced by co-transfecting the package vector expressing *gag* and *vsvg* genes with the indicated plasmids into HEK293T cells and harvested 48 h after transfection. A549 cells were transduced with the retrovirus in the presence of 8 μg/ml polybrene. The shRNA plasmids produced retrovirus infected cells were selected in puromycin (2 μg/ml) for knocking down and the pLHCX (Clontech) plasmids containing two silent nucleotide substitutions in the sequence corresponding to the shRNA-targeted region produced retrovirus infected cells were selected in hygromycin (350 mg/ml) for putting back. After 7-12 days of selection, the expression levels of PKM2 were determined by western blot. The sequences of shRNA and control RNA were reported previously [7].

### Cell proliferation and xenograft studies

5×10^4^ indicated stable cells were seeded in triplicate in 6-well plates and cell numbers were counted every day over a 4-day period. Nude mice (nu/nu, male 6 to 7-week-old) were injected subcutaneously with 5×10^6^ A549 PKM2 knocking-down and wild-type or K498E mutant putting-back stable cells. Seven weeks later, the tumors were harvested, and the volume and weight of tumors were measured.

### Generating site specific succinylation antibody

PKM2 K498 site-specific succinylation antibody was generated by using a human PKM2 succinylated peptide (DLRVNFAMNVGK^suc^ARGFFKKGDVVIVL) as an antigen. After purifying the antibodies with excess unmodified peptides, antibodies recognizing site specific succinylation were enriched by biotin labeled PKM2 succinylated peptides. The specificity of site specific succinylation antibody was verified by western blot.

### Statistical analysis

Data were analyzed using Student's t test, and statistical significance was defined as *P < 0.05.

## SUPPLEMENTARY MATERIALS FIGURES


