# Supplementary material for: Desuccinylation of pyruvate kinase M2 by SIRT5 contributes to antioxidant response and tumor growth
Source: Oncotarget. 2016 Dec 28;8(4):6984–93. doi: 10.18632/oncotarget.14346 (PMC5351684; doi:10.18632/oncotarget.14346)
Supplement: Supplementary file 1 [file oncotarget-08-6984-s001.pdf]

Desuccinylation of pyruvate kinase M2 by SIRT5 contributes to antioxidant response and tumor growth

SUPPLEMENTARY FIGURES

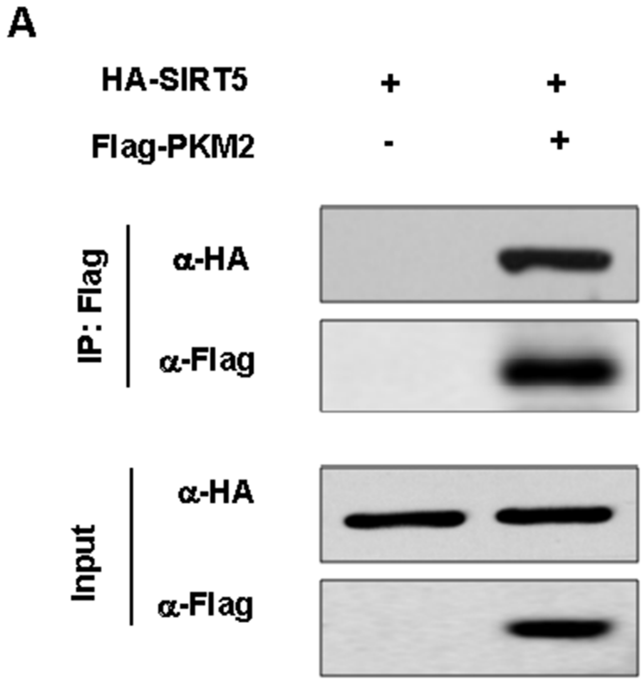

Supplementary Figure 1: A. Exogenous SIRT5 binds to PKM2. 293T cells were transfected with indicated plasmids and the binding of PKM2 to SIRT5 was examined by IP and western blot.

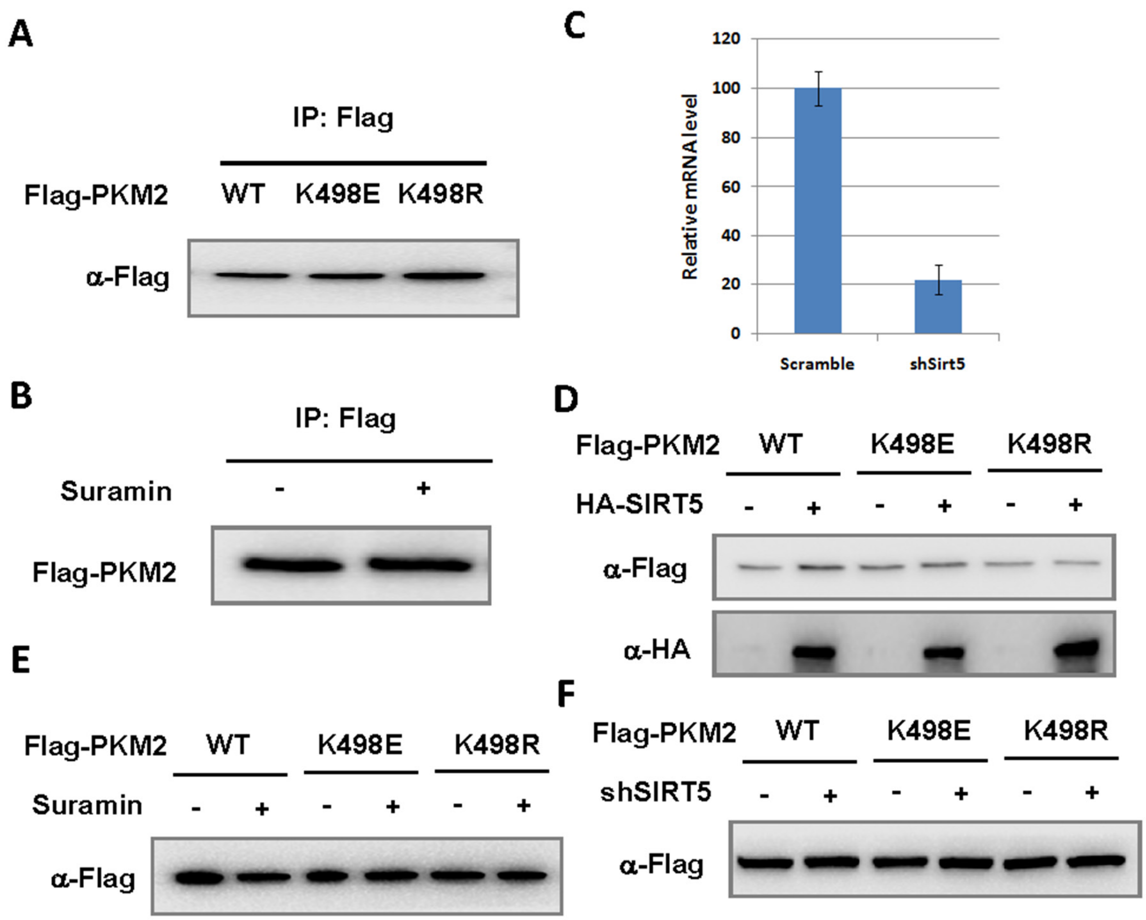

Supplementary Figure 2: A. Flag-tagged wild-type and mutant PKM2 proteins were expressed in 293T cells and purified by immunoprecipitation. The relative enzyme activities were presented in Figure 2A. PKM2 protein levels were analyzed by western blot. **B.** 293T cells were transfected with Flag-PKM2, followed by treatment with Suramin (40 uM). Flag-PKM2 was immunoprecipitated and activity was measured. Result was shown in Figure 2B. PKM2 protein levels were analyzed by western blot. **C.** SIRT5 knockdown efficiency in 293T cells was determined by q-PCR. Error bars represent ±SD for triplicate experiments. **D.** Wild-type or mutant PKM2 were co-expressed in 293T cells with or without SIRT5 respectively and purified with Flag beads, followed by enzyme assay. Result was shown in Figure 2D. PKM2 and SIRT5 protein levels were analyzed by western blot. **E.** Wild-type or mutant PKM2 was transfected into 293T cells with or without the treatment of Suramin (40 uM). Proteins were purified with Flag beads, followed by enzyme assay. Result was shown in Figure 2E. PKM2 protein levels were analyzed by western blot. **F.** Wild-type or mutant PKM2 were transfected into scramble and SIRT5 knocking down 293T cells respectively and proteins were purified with Flag beads, followed by enzyme assay. Result was shown in Figure 2E. SIRT5 knocking down efficiency was verified in Supplementary Figure 2C. PKM2 protein levels were analyzed by western blot.

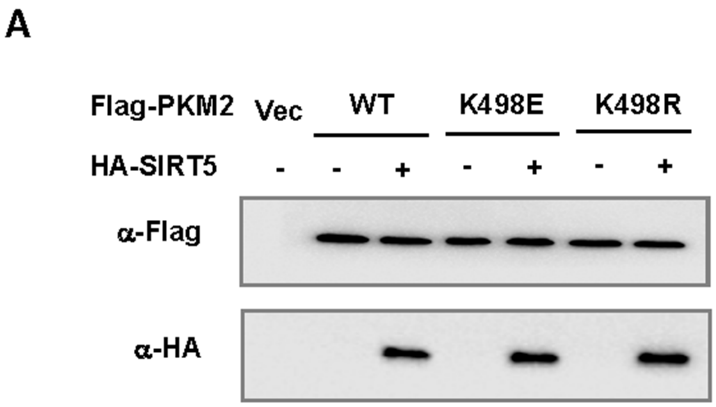

Supplementary Figure 3: A. Wild-type or mutant PKM2 were co-expressed in 293T cells with or without SIRT5 respectively and DCF staining was performed to measure ROS levels. Result was shown in Figure 3E. PKM2 and SIRT5 protein levels were analyzed by western blot.

A

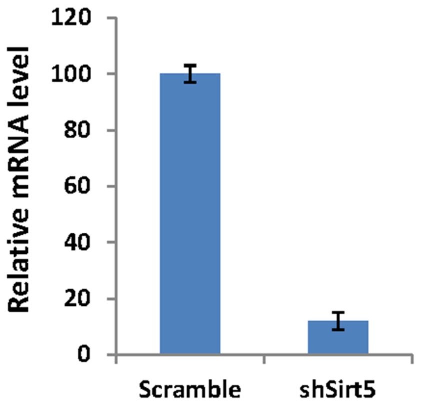

Supplementary Figure 4: A. SIRT5 was knocked down in A549 cells. Knockdown efficiency was determined by q-PCR. Error bars represent ±SD for triplicate experiments.
